# Interim U.S. Guidance for Monitoring and Movement of Persons with Potential Ebola Virus Exposure

**Published:** 2014-10-31

On October 27, 2014, CDC released Interim U.S. Guidance for Monitoring and Movement of Persons with Potential Ebola Virus Exposure (available at http://www.cdc.gov/vhf/ebola/exposure/monitoring-and-movement-of-persons-with-exposure.html). This updated guidance focuses on strengthening the monitoring of persons potentially exposed to Ebola and evaluating their intended travel, including the application of movement restrictions when indicated. This interim guidance has been updated by establishing a “low (but not zero) risk” category; adding a “no identifiable risk” category; modifying the recommended public health actions in the “high risk,” “some risk,” and “low (but not zero) risk” categories; and adding recommendations for specific groups and settings.

Through these changes, CDC and state and local health departments seek to support persons who might have been exposed to Ebola, while also continuing to stop Ebola at its source in West Africa. These changes will help ensure that health care workers returning to the United States from West Africa are monitored, any symptoms they might develop are quickly identified, and that a system is in place to recognize when they need to be routed to care. These actions will better protect potentially exposed individuals and the U.S. public as a whole. A fact sheet regarding the new interim guidance is available at http://www.cdc.gov/media/releases/2014/fs1027-monitoring-symptoms-controlling-movement.html.

